# Comparative study on chloroplast genome of *Tamarix* species

**DOI:** 10.1002/ece3.70353

**Published:** 2024-10-01

**Authors:** Yanlei Liu, Kuo Ding, Lixiong Liang, Zhan Zhang, Kai Chen, Haiwen Li

**Affiliations:** ^1^ School of Landscape and Ecological Engineering Hebei University of Engineering Handan China; ^2^ Bingtuan Xingxin Vocational and Technical College Tiemenguan China; ^3^ College of Life Sciences and Technology Tarim University Alar China

**Keywords:** chloroplast genome, identification, next‐generation sequencing, phylogeny, *Tamarix*

## Abstract

Tamaricaceae comprises about 120 species and has a long evolutionary history, *Tamarix* Linn accounts for approximately 75% of the total species in this family. It is the most widely distributed and diverse genus in the family. They have important ecological significance for transforming deserts and improving climate conditions. However, *Tamarix* is the most poorly classified genera among flowering plants owing to its large variability and high susceptibility to interspecific hybridization. In this study, the complete chloroplast genomes of three *Tamarix* species and one draft chloroplast genome were obtained in this study. Combined with eight chloroplast genomes deposited in GenBank, complete chloroplast sequences of 12 *Tamarix* species were used for further analysis. There are 176 non‐SSR‐related indels and 681 non‐indel‐related SSRs in the 12 *Tamarix* chloroplast genomes. The mononucleotide SSRs are the most prevalent among all types of SSRs. The mVISTA results indicate high sequence similarities across the chloroplast genome, suggesting that the chloroplast genomes are highly conserved, except for sample *Tamarix androssowii* (ENC850343). The IR regions and the coding regions are more conserved than the single‐copy and noncoding regions. The *trnF‐ndhJ*, *ndhC‐trnM‐*CAU, *ycf1*, and *trnL‐*UAG*‐ndhF* regions are the most variable and have higher variability than those of the universal DNA markers. Finally, the first phylogenetic tree of Tamaricaceae was constructed which confirmed the monophyly of *Tamarix* in Tamaricaceae. The first phylogenetic tree of *Tamarix* was based on the complete chloroplast genome to date, the changes in branch length and support rate can potentially help us clarify the phylogenetic relationships of *Tamarix*. All the obtained genetic resources will facilitate future studies in population genetics, species identification, and conservation biology of *Tamarix*.

## Graphical abstract

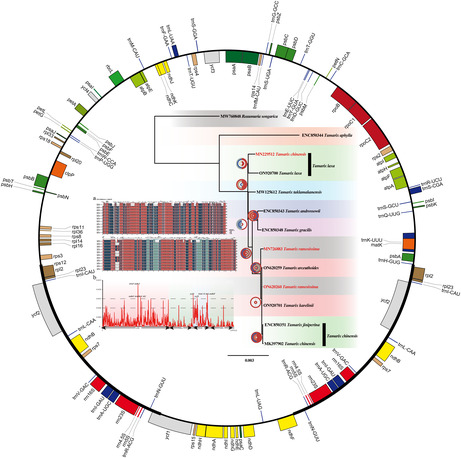

## 1 INTRODUCTION

Tamaricaceae has a long evolutionary history that originated in the Tertiary period. There are four genera and approximately 120 species in the world, mainly distributed in grasslands and desert areas of the Old World (Zhang, 2005). Among them, *Tamarix* Linn has over 90 species worldwide, accounting for approximately 75% of the total number of species in Tamaricaceae. It is the most widely distributed and diverse genus in Tamaricaceae, which is a natural group (Wu et al., 2007). Among them, 18 species and one variety were found in China, accounting for approximately 20% of the world's *Tamarix* species. *Tamarix* species in China are mainly distributed in northwest China, Inner Mongolia, and North China, especially in Xinjiang Province. They are primarily found in saline or low‐humidity sand areas in desert or semi‐desert regions (Baum, 1978; Gaskin, 2003; Gaskin & Schaal, 2003; Wu et al., 2007). The plants of this genus are mainly xerophytes, and some are excellent tree species for wind prevention, sand fixation, afforestation, and soil and water conservation which have important ecological significance for transforming deserts and improving climate conditions. Moreover, *Tamarix* plants are the main source of fuel, fertilizer, and food in desert areas (Bahramsoltani et al., 2020). Species in *Tamarix* can also be used as dyes and spices, and the entire plant can be used in Chinese medicine (Chen et al., 2018).

The importance of *Tamarix* species in desert areas is known, and research on their adaptation mechanisms to harsh environments, such as low temperature, drought, and high salinity, has continued. Researchers have studied salt tolerance (Wang et al., 2010, 2021; Zhang & Xu, 1993), drought resistance (Lee, 2022; Liu et al., 2014; Sun et al., 2022), seed germination mechanisms and characteristics (Liu et al., 2014; Waisel, 1960; Yan et al., 2011), environmental adaptation mechanisms (Liu et al., 2023a; Sghaier et al., 2015; Wei et al., 2020), and other aspects of *Tamarix*. However, previous studies have also shown that there are differences in the performance of *Tamarix* species in adapting to extreme climate environments.

Among *Tamarix* species, determining which species are most adaptable to local ecological environments remains challenging. This difficulty stems primarily from the poor classification of the genera *Tamarix*, which is one of the most poorly classified genera among flowering plants owing to its extensive variability and high susceptibility to interspecific hybridization, with over 200 species names appearing in the literature (Baum, 1978). This taxonomic confusion complicates the analysis of geographical flora and the application of species in ecological reconstruction projects (Boissier, 1867). Furthermore, the lack of reliable fossil records has resulted in limited comprehensive and systematic research on the origin, distribution, and geographical spread of this genus. Baum's monograph provides only a brief discussion on the origin and distribution of *Tamarix* (Baum, 1978). In 1852, 51 species of *Tamarix* were identified, with 21 were recorded for the first time (von Bunge, 1852). By 1895, the number of recorded *Tamarix* species had increased to 67 (Niedenzu, 1895), and this number rose to 78 in subsequent editions. In the nearly 100 years that followed, new *Tamarix* species continued to be described. Eventually, 200 species of *Tamarix* were classified into 54 species, divided into three groups and nine series (Baum, 1978). Many of these species were either treated as synonyms, reduced to varieties, or mistakenly identified (Baum, 1978). This effort significantly clarified the chaotic classification and unclear number of species within *Tamarix*. However, Baum's work was based on extensive research on wax leaf specimens and had limited data from regions such as China, Mongolia, and Pakistan. Subsequent studies, including Qaiser et al.'s research in Pakistan in 1981 and the publication of flora from China, Mongolia, and other regions, have further refined the taxonomic grouping of this genus. The current classification includes nine series with 68 species and seven varieties (Qaiser & Perveen, 2004). These findings highlight the difficulty in determining the classification and accurate identification of *Tamarix* species using conventional taxonomic features.

With the advent of next‐generation sequencing (NGS), the era of omics has arrived, with a large amount of genomic and transcriptome data being produced for multidisciplinary research (Liu et al., 2023b). By using complete chloroplast genomes, researchers have made great progress in resolving the unclear phylogenic relationship of closely related species and species identification, some researchers even advocated treating the complete chloroplast genome as a super‐barcode for plant species identification which shines a bright light on phylogeny and identification problems in *Tamarix* species (CBOL Plant Working Group et al., 2009; Cheng et al., 2016; Dong et al., 2022; Kress, 2017; Li et al., 2015; Wang et al., 2023; Yu et al., 2011; Zhang et al., 2021). In this study, we aim to provide support for taxonomic research and precise identification of *Tamarix* species by comparing the differences in chloroplast genomes among *Tamarix* species for the first time.

## 2 MATERIALS AND METHODS

### 2.1 Plant materials and DNA extraction

Four *Tamarix* species leaves were obtained from the Herbarium of the Institute of Botany, Chinese Academy of Sciences (PE) with specimen barcode numbers ENC850343 (PE01177239), ENC850344 (PE01177243), ENC850348 (PE02068788), and ENC850351 (PE01983734). Experts in the field had previously identified these materials morphologically with high accuracy (more details are listed in Table S1). Total DNA was extracted using the method described by Li et al. (2013) and purified with a Wizard DNA cleanup system (Promega, Madison, WI, USA). The quality of the DNA was assessed via spectrophotometry, and its integrity was evaluated using a 1.5% (w/v) agarose gel.

### 2.2 Sequencing, assembly, and annotation

The total DNA was fragmented into 350 bp fragments using ultrasound (Covaris M220). A paired‐end library was constructed using an NEB Next UltraTM DNA library prep kit, and PE150 (paired‐end 150 bp) sequencing was conducted on the Illumina HiSeq 2500 platform. Quality control and filtering of low‐quality reads were performed using the default parameters of the NGS QC toolkit. Contigs were assembled from the high‐quality paired‐end reads using the SPAdes 3.6.1 program (K‐mer = 95) (Bankevich et al., 2012). The chloroplast genome contigs were then selected using the Blast2.6^+^ program, with the chloroplast genome of *Tamarix chinensis* (MK397902) as a reference (Altschul et al., 1990). The selected contigs were subsequently assembled using Sequencher v5.10. To verify the accuracy of the assembly, all reads were mapped to the assembled chloroplast genome sequence using Geneious Prime v2022.2.1 (Kearse et al., 2012). The complete chloroplast genome sequences were annotated with Plann.pl. using *Tamarix chinensis* (MK397902) as a reference (Huang & Cronk, 2015), and a ring diagram was created by using Organellar Genome DRAW v1.1 (https://chlorobox.mpimp‐golm.mpg.de/OGDraw.html) and modified using Adobe Illustrator CC 2018 (Lohse et al., 2007).

### 2.3 Analysis of microstructural mutation events

To understand the microstructural mutations within the chloroplast genomes of *Tamarix* species, including variable mutation sites, parsimony information sites, and simple sequence repeats (SSR), 12 chloroplast genomes (eight of which were downloaded from NCBI, as detailed in Table S1) were first aligned using MAFFT V7 online software (Katoh & Standley, 2013), and then manually adjusted using BioEdit v7 (Hall et al., 2011). Variable mutation sites and parsimony information sites in the chloroplast genome were determined using MEGA 10.0.5 (Kumar et al., 2008). SSRs were predicted using the GMATA software package (Genome wide Microsatellite Analyzing Tool Package), with parameters set at greater than or equal to 10 repeat units for mononucleotide, greater than or equal to five repeat units for dinucleotide, greater than or equal to four repeat units for trinucleotide, and greater than or equal to three repeat units for tetranucleotide, pentanucleotide, and hexanucleotide SSRs (Wang & Wang, 2016). Based on the aligned sequence matrix, indels were manually validated and categorized into SSR‐related and non‐SSR‐related (normal) indels using Sequencher V5.4.5. *T. aphylla* (ENC850344) was used as the reference to determine the size and position of the indel events.

#### 2.3.1 Hotspot identification

To find the most variable regions in the chloroplast genome of *Tamarix*, a comparison of the complete chloroplast genomes of *Tamarix* was conducted using the mVISTA program (http://genome.lbl.gov/vista/mvista/submit.shtml) in Shuffle‐LAGAN mode, with the sequence of *T. aphylla* serving as the reference. Nucleotide diversity within the chloroplast genome was calculated via sliding window analysis using DnaSP v6.12.03 software (Librado & Rozas, 2009), with a window length of 600 bp and a step size of 200 bp.

#### 2.3.2 Phylogenetic reconstruction

Two analyses were performed to reconstruct phylogeny. The first analysis aimed to establish the monophyly of *Tamarix*. All available complete chloroplast genome data for the Tamaricaceae were retrieved from public databases. After data deduplication, 21 complete chloroplast genome sequences from Tamaricaceae species (including eight *Myricaria* species, 11 *Tamarix* species, and two *Reaumuria* species) were used for phylogenetic reconstruction. Since our focus was solely on determining the monophyly of *Tamarix*, maximum likelihood (ML) method was employed. The optimal model TVM + F + I + G4 was determined by ModelFinder based on the BIC standard (recommended by the software) (Kalyaanamoorthy et al., 2017). ML calculations were performed using the IQ tree software (Nguyen et al., 2015), with 1000 bootstrap replicates.

For the phylogenetic reconstruction within *Tamarix*, 12 chloroplast genome sequences were used, including eight *Tamarix* samples from GenBank (Table S1). All chloroplast genome sequences were aligned using the MAFFT online version (https://mafft.cbrc.jp/alignment/server/), and ambiguous alignment regions were trimmed using Gblocks 0.91b (Castresana, 2000). Phylogenetic analysis was conducted using both the ML and Bayesian inference (BI) methods. The optimal model TVM + F + I + G4 was calculated by ModelFinder based on the BIC standard (recommended by the software) (Kalyaanamoorthy et al., 2017). ML calculations were performed using the IQ tree software (Nguyen et al., 2015), with 1000 bootstrap replicates. BI of the phylogenies was implemented using the MrBayes program (Ronquist et al., 2012). The Markov chain Monte Carlo (MCMC) analysis was run for 10,000,000 generations, with trees sampled every 1000 generations. The initial 25% of samples were discarded as burn‐in. Finally, the average standard deviation of split frequencies was verified to be less than 0.01.

## 3 RESULTS

### 3.1 Chloroplast genome sequencing and features of *Tamarix* species

In this study, complete chloroplast genomes for three *Tamarix* species and one draft chloroplast genome (composed of concatenated reliable contig data but with gaps in some regions) were obtained. Combined with eight chloroplast genome samples from GenBank, 12 *Tamarix* species were used for further analysis (Table S1). The total chloroplast genome size ranged from 155,977 bp (*T. ramosissima* ON620260) to 156,190 bp (*T. chinensis* MN229512). *Tamarix* chloroplast genome was found to have a typical quadripartite structure, including a pair of IR regions (Inverted Repeat regions, 26,470–26,575 bp), LSC regions (Large Single Copy regions, 84,745–85,060 bp), and SSC regions (Small Single Copy regions, 17,871–18,262 bp). The average GC content was determined to be 36.5% across the total chloroplast genome, with 42.4% in IR regions, 34.2% in LSC regions, and 29.6% in SSC regions.

**FIGURE 1:**
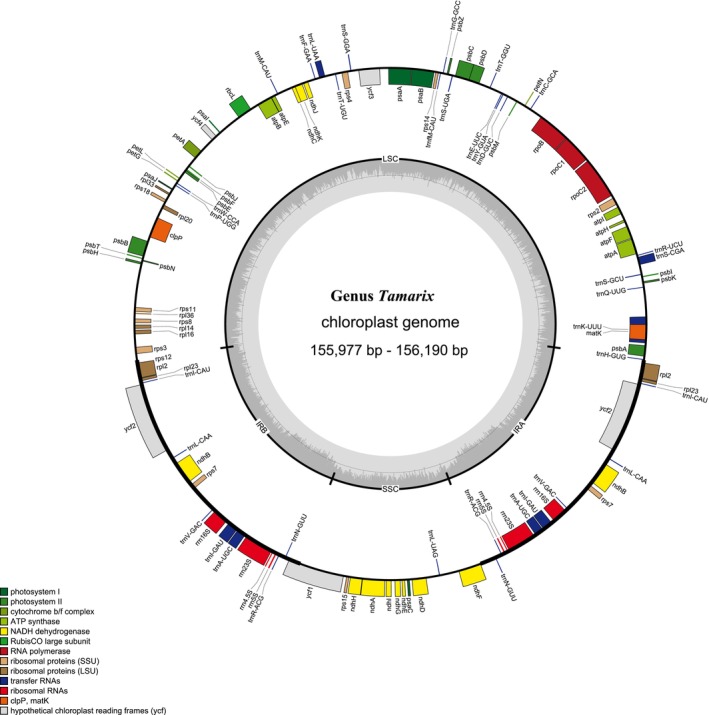
Physical map of chloroplast complete genome of *Tamarix* species.

The complete chloroplast genome of *Tamarix* species includes 120 to 130 genes, encompassing 76 to 85 protein‐coding genes, 36 to 37 transfer RNA genes, and eight ribosomal RNA genes (Figure 1, Table S1). Six protein‐coding genes (*ndhB*, *rpl23, rps7*, *rps12*, *ycf2*, and *rpl2*), seven tRNA genes (*trnI*‐CAU, *trnL*‐CAA, *trnV*‐GAC, *trnI*‐GAU, *trnA*‐UGC, *trnR*‐ACG, and *trnN‐GUU*) and all four rRNA genes are duplicated in the IR regions. Fourteen genes (*atpF*, *rpoC1*, *ndhB*, *petB*, *rpl2*, *ndhA*, *rps12*, *rps16*, *trnA*‐UGC, *trnI*‐GAU, *trnK*‐UUU, *trnL*‐UAA, *trnG*‐GCC, and *trnV*‐UAC) contain a single intron, and two genes (*clpP* and *ycf3*) have two introns. The *rps12* gene is a trans‐spliced gene with the 5′‐end located in the LSC region and the 3′ end located in the IR region. The *trnK*‐UUU gene has the largest intron, which contains the *matK* gene.

### 3.2 Indels and SSRs

**FIGURE 2:**
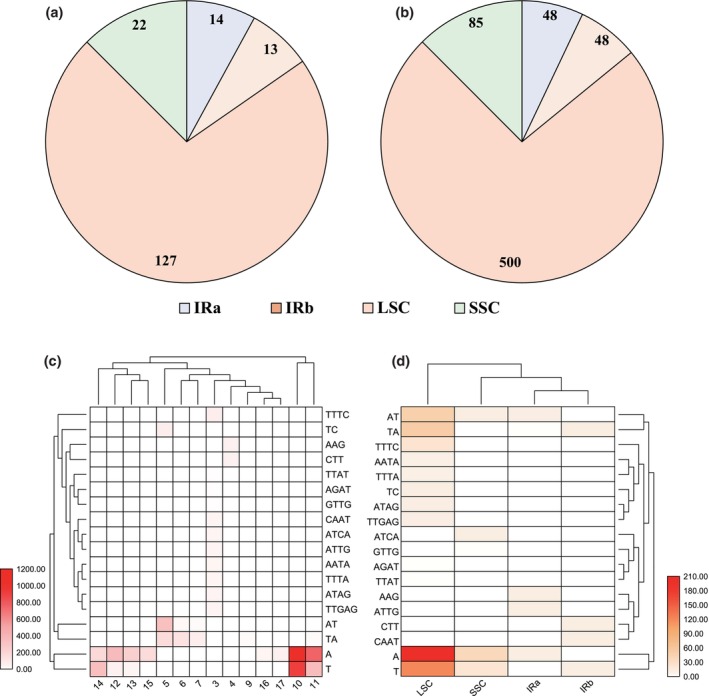
Indel (a) and SSR (b) situation in each region in the complete chloroplast genome region. SSR situations among *Tamarix* species. (c) The occurrence frequency and repetitive unit situation of different SSR types in *Tamarix* group. (d) The distribution frequency of different types of SSRs in each partition.

A total of 176 non‐SSR (Simple Sequence Repeat) related indels were identified in the 12 *Tamarix* chloroplast genomes, comprising 99 insertions and 77 deletions. The number of insertions in the LSC, IRb, SSC, and IRa regions was 68, 8, 14, and 9, respectively, while the number of deletions in these regions was 59, 5, 8, and 5, respectively (Figure 2a). Additionally, 681 non‐indel‐related SSRs were identified, with the SSR counts in the LSC, IRb, SSC, and IRa regions being 500, 48, 85, and 48, respectively (Figure 2b).

Through clustering analysis, mononucleotide SSRs were found to exist exclusively in the A/T type, which had the highest number of repeats among all SSR types, accounting for 36.7% and 22.8% of the total SSR number (Figure 2c). Ten and eleven repeats were the primary SSR types, predominantly distributed in the LSC region, followed by the SSC region (Figure 2d). The main types of dinucleotide SSRs were AT (71), TA (65), and TC (12), with five repeats being the most common type. These dinucleotide SSRs were primarily distributed in the LSC region, comprising 75.7% of the total (Figure 2d). Additionally, trinucleotide SSRs appeared 24 times, tetranucleotide SSRs appeared 93 times, and pentanucleotide SSRs appeared 11 times. Trinucleotide SSRs were mainly found in the IR region, while tetranucleotide SSRs were mainly found in the LSC region (56 of 93 occurrences), with three repeats being the most common frequency (Figure 2d).

### 3.3 Sequence divergence and hotspots

**FIGURE 3:**
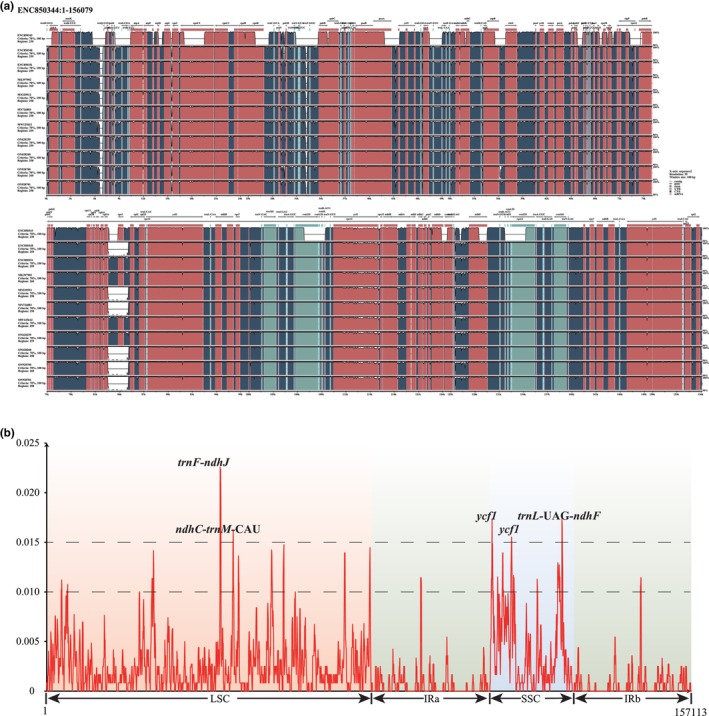
mVISTA results of 12 *Tamarix* CPG samples (a). Nucleotide diversity among *Tamarix* species (b). The x‐axis represents the position in the chloroplast genome, and the y‐axis represents the value of nucleotide polymorphism. LSC is represented by a light red background, IR regions are indicated by a light blue background, and SSC is represented by a light blue background.

A comparative analysis based on mVISTA was performed on the 12 chloroplast genomes of *Tamarix* to determine the level of divergence (Figure 3a). The results indicate high sequence similarities across the chloroplast genome, suggesting that they are highly conserved, with the exception of sample *T. androssowii* (ENC850343), which is a draft chloroplast genome. The IR and coding regions were found to be more conserved than the single‐copy and noncoding regions.

Additionally, the nucleotide diversity of the 12 *Tamarix* chloroplast genomes was compared. These genomes were aligned into a matrix of 157,207 bp, revealing an average nucleotide diversity value of 0.0021. The IR regions exhibited the lowest nucleotide diversity, while the SSC regions showed the highest diversity. DnaSP v6.12.03 was used to measure nucleotide diversity and identify mutation hotspot regions in the complete *Tamarix* chloroplast genomes (Figure 3b). The *trnF‐ndhJ* region had the highest Pi values (Pi = 0.021), followed by three other regions (Pi > 0.015): ndhC‐*trnM*‐CAU in the LSC region, ycf1, and *trnL*‐UAG*‐ndhF* in the SSC region. The variability of the four identified mutation hotspot regions was tested alongside three universal chloroplast DNA barcodes (*matK*, *rbcL*, and *trnH‐psbA*). The newly identified markers showed higher variability among *Tamarix* species compared to the universal DNA barcodes.

### 3.4 Phylogenetic analysis

**FIGURE 4:**
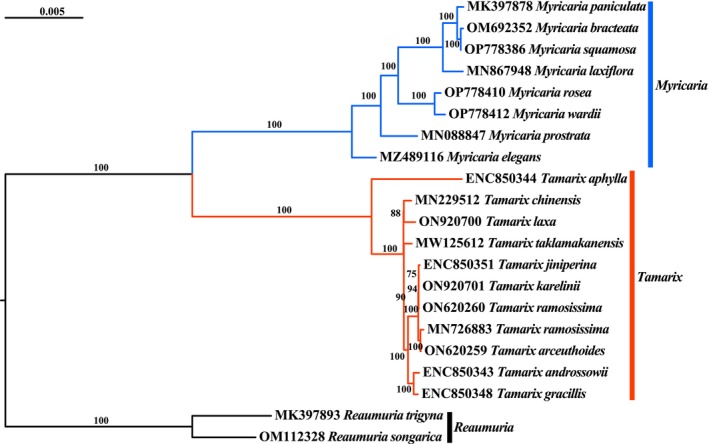
Phylogenetic relationship of Tamaricaceae. ML tree of Tamaricaceae based on the complete chloroplast genome dataset. Different genera are indicated by different line colors. The vertical lines on the right delineate the sample range for each genus. The numbers on the horizontal lines represent the support values for the different branches.

From the phylogenetic results of the Tamaricaceae, the species are mainly divided into three major groups: *Myricaria* species, *Tamarix* species, and *Reaumuria* species each form a distinct group, with *Tamarix* being a clearly monophyletic group (Figure 4).

**FIGURE 5:**
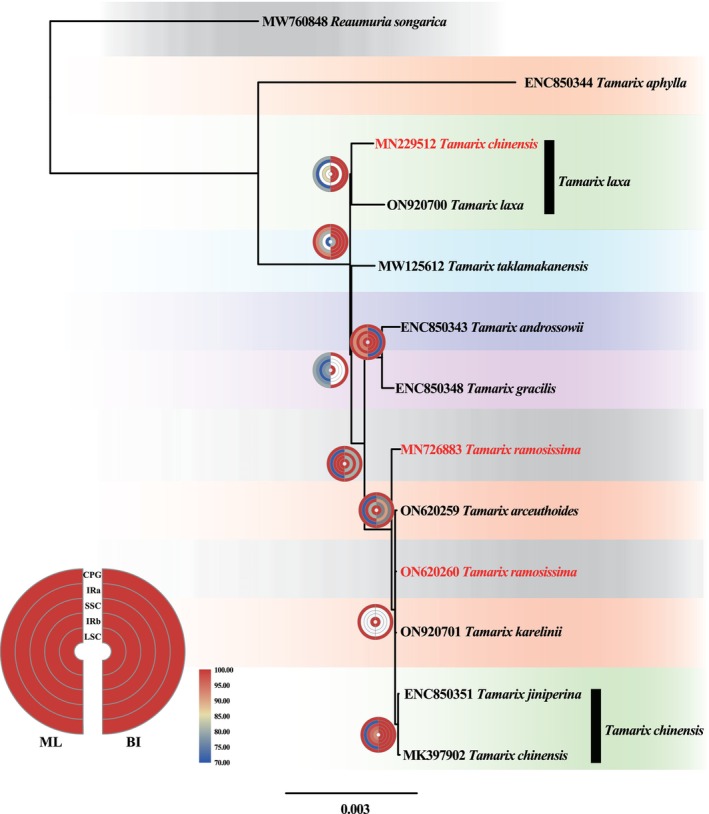
Phylogenetic relationship of *Tamarix*. Different species in the figure are marked with different background colors, and samples that might have issues are highlighted in red font. On the right side of the semicircle, the left side shows the ML support values, while the right side displays the BI posterior probabilities. The semicircle represents support values or posterior probabilities for LSC, IRb, SSC, IRa, and the complete chloroplast genome datasets from inner to outer. The magnitude of support or posterior probabilities is displayed using a blue‐yellow‐red gradient percentage scale.

Using the complete chloroplast sequences, phylogenetic relationships of the 12 *Tamarix* species were constructed. The topologies of the ML and BI results were essentially consistent and included five datasets (complete chloroplast genome, LSC, IRb, SSC, and IRa; Figure 5). From the terminal to the base branches of the phylogenetic trees, the first group is the cluster of *T. jiniperina* and *T. chinensis*, with a support rate of 100%. Upon performing a literature review, *T. jiniperina* was found to be a former name for *T. chinensis*, however, another *T. chinensis* (MN229512) sample in the public database is not clustered together with these two *T. chinensis* samples. Instead, this sample is clustered with *T. laxa* (ON920700) which may indicate the wrong identification of this sample. The two *T. chinensis* samples together clustered with *T. karelinii* (ON920701) which indicates a close phylogenic relationship between *T. chinensis* and *T. karelinii*. *T. chinensis, T. karelinii, T. ramosissima* (ON620260), *T. arcuthoids* (ON620259), and *T. ramosissima* (MN726883) form a large branch, which is a sister group to the group containing *T. gracilis* and *T. androssowii*. *T. taklamakanensis* is a sister group to the species found at the terminal branches of the phylogenetic tree. The group they collectively form and the branch of *T. laxa* are sister taxa. *T. aphylla* is the basal group of *Tamarix* (Figure 5). Notably, the two samples of *T. ramosissima* did not clustered together.

## 4 DISCUSSION

### 4.1 The chloroplast genome of *Tamarix*

In this study, the chloroplast genomes of four *Tamarix* species were sequenced by NGS methods. Combined with eight complete chloroplast genome data deposited in NCBI, the chloroplast genome size ranges from 155,977 bp (*T. ramosissima*) to 156,190 bp (*T. chinensis*). All species have 120 to 130 genes, including 76 to 85 protein‐coding genes, 36–37 transfer RNA genes, and eight ribosomal RNA genes, in the chloroplast genome. In this study, the *ycf15* and *ycf68* genes were not annotated because of several internal stop codons in these regions which also appeared in the former study (Lu et al., 2017). The chloroplast genome is conserved similarly to the majority of plants; no rearrangement events were detected in any of *Tamarix* species. The mVISTA results and nucleotide diversity tests indicate high similarities between the chloroplast genomes, implying that the divergence of *Tamarix* chloroplast genome is lower than that of other species which is the same as former studies (Li et al., 2018; Song et al., 2017; Xu et al., 2017). One hundred and seventy‐six non‐SSR‐related indels and 681 non‐indel‐related SSRs were found in the 12 *Tamarix* chloroplast genomes. Indels are another important class of genetic variation. In SSR‐related indels, polymerase slippage results in the addition or deletion of short spans of sequences that repeat at one side of the region flanking the indels which is the same as the former study (Graham et al., 2000). The majority of the SSR‐related indels were primarily detected in the A/T regions which is also confirmed by Gandhi's study (Gandhi et al., 2010). Intramolecular recombination and hairpins or the stem‐loop secondary structure cause the majority of the non‐SSR‐related mutations which is the same as Song's study (Song et al., 2019). In most cases, non‐SSR‐related indels are more frequent than SSR‐related indels (Song et al., 2019). In the genome of *Tamarix*, non‐SSR‐related indels are over two‐fold more frequent than SSR‐related indels. Nucleotide divergence is significantly correlated with the size and abundance of the nearby indels which is also supported by multiple studies (Ahmed et al., 2012; Hollister et al., 2010; Tian et al., 2008), which indicates that indels may be associated with mutation hotspots.

### 4.2 Phylogenetic relationships

The phylogenetic results of the Tamaricaceae confirm the monophyly of *Tamarix* which is the same as taxonomy treatment in traditional taxonomy (Figure 4). However, owing to the low genetic divergence and similar morphology, the systematic relationships within *Tamarix* remain unclear. A former study proved that the use of several chloroplast markers or nuclear markers, such as the internal transcribed spacer (ITS), *trnL‐ndhF*, and *rbcL*, for phylogenetic resolution is insufficient for drawing firm conclusions about the inter‐species relationships in *Tamarix* (Mayonde et al., 2015; Sun et al., 2016; Villar et al., 2019). Up to now, complete chloroplast genomes have not been used to study the phylogenetic relationships within *Tamarix* largely due to the difficulty in accurately distinguishing *Tamarix* species using morphological traits. Fortunately, large‐scale application of next‐generation sequencing (NGS) technology has enhanced the ability to sequence complete chloroplast genomes, resulting in the resolution of closely related species using complete chloroplast sequences (Cai et al., 2021; Dong et al., 2018; Viljoen et al., 2018). In this study, the complete chloroplast sequences were first used to assess the phylogenetic relationships within *Tamarix*. Finally, the first robust phylogenomic tree based on the complete chloroplast genome of *Tamarix* was obtained. Based on the chloroplast genome, the phylogenetic relationships among *Tamarix* species still exhibit very short branch lengths and minimal differences at the terminal taxa (Figure 5). Although complete chloroplast genome data were used to study the relationships among *Tamarix* species, issues such as the inability to group samples of the same species together are still being encountered [*T. ramosissima* (ON620260) and *T. ramosissima* (MN726883)], misidentification of species [*T. chinensis* (MN229512) is a sample that has been incorrectly identified; its correct name should be *T. laxa*] and inconsistent species naming (*T. jiniperina* and *T. chinensis*). Hence, it can be indirectly understood that the classification of *Tamarix* species is not easy through the identification errors found in the phylogenetic tree samples. Fortunately, through the construction of a phylogenetic tree using the complete chloroplast genome, the classification of *Tamarix* species could be achieved based on branch length and support rate. Thus, this is a successful attempt to reconstruct phylogenetic relationships among *Tamarix* species using the complete chloroplast genome. Unfortunately, it is difficult to distinguish *Tamarix* in terms of morphology. However, this study only included nine species and 12 samples. We are currently obtaining reliable *Tamarix* samples through various legal databases from around the world. To solve the phylogenetic relationship and species classification of *Tamarix*, it still needs a larger sampling size, includes as many species as possible, and needs international cooperation.

### 4.3 Potential highly variable chloroplast barcodes

An increasing number of case studies indicate that universal DNA barcodes have lower divergence and poor discriminatory power (Dong et al., 2012). In *Tamarix*, these regions lack variability and may lead to unsuccessful identification and confusing relationships among species (Figure 3b). *Tamarix* species are an important building species in desert areas that have significant value for the protection and reconstruction of the ecological environment in these areas. However, the lack of genomic resources for *Tamarix* species is the main obstacle to taxonomy, genetics, identification, and conservation. Chloroplast genome sequences provide an opportunity to determine the genome evolution and generate valuable genetic resources for further studies. The mutation events in the chloroplast genome are not universally randomly distributed within the sequence and are concentrated in certain regions forming “hotspot” regions (Dong et al., 2012). Comparison of the chloroplast genome sequences is an effective strategy to identify the mutation hotspots, and these highly variable regions can be used as specific DNA barcodes. In this study, four hypervariable regions were identified, including *trnF‐ndhJ*, *ndhC‐trnM‐*CAU, *trnL‐*UAG*‐ndhF*, and *ycf1*. However, these markers have not been extensively used in plant phylogeny and DNA barcoding. Before the emergence of NGS technology, some researchers found that *ycf1* is a candidate gene with high resolution in angiosperms (Dong et al., 2015), but its length is too long, and universal primers are difficult to design. The sequence length span of the other three regions is relatively short at approximately 400 bp (base pair). This study did not design specific amplification primers for these four regions. The design of primers requires a large amount of data from different species as support for its universality. Therefore, this study only proposes these four highly variable regions, which can also be obtained by NGS sequencing.

## 5 CONCLUSIONS

In this study, the complete chloroplast genomes of four *Tamarix* species were sequenced and assembled, the first robust phylogenetic relationship of *Tamarix* was constructed, and valuable genomic resources were provided for this genus. Meanwhile, the comparative analysis of chloroplast genomes generated variable regions, which could be used as specific DNA barcodes. All the obtained genetic resources will facilitate future studies on the population genetics, species identification, and conservation biology of *Tamarix*.

## AUTHOR CONTRIBUTIONS

**Yanlei Liu:** Conceptualization (equal); data curation (equal); formal analysis (equal); funding acquisition (equal); investigation (equal); methodology (equal); resources (equal); software (equal); visualization (equal); writing – original draft (equal); writing – review and editing (equal). **Kuo Ding:** Conceptualization (equal); data curation (equal); formal analysis (equal); writing – original draft (equal). **Lixiong Liang:** Investigation (equal); resources (equal); visualization (equal). **Zhan Zhang:** Conceptualization (equal); data curation (equal); resources (equal); visualization (equal); writing – original draft (equal). **Kai Chen:** Supervision (equal); visualization (equal); writing – original draft (equal); writing – review and editing (equal). **Haiwen Li:** Supervision (equal); writing – original draft (equal); writing – review and editing (equal).

## FUNDING INFORMATION

Natural Science Foundation of Hebei Province (C2022402017), Project YJZDKT202315, and Natural Science Foundation of China (NSFC32101496).

## CONFLICT OF INTEREST STATEMENT

The authors declare no conflict of interest.

## CONSENT FOR PUBLICATION

Not Applicable.

## Supporting information


**TABLE S1.** The basic plastomes information of 12 *Tamarix* samples.

## Data Availability

All four CPG data were deposited in GenBank and the accession numbers were OR619632 for ENC850343, OR619629 for ENC850344, OR619630 for ENC850348, and OR619631 for ENC850351. All necessary data files were provided to editors and reviewers.
