# Supplementary material for: Comparative study on chloroplast genome of Tamarix species
Source: Ecol Evol. 2024 Oct 1;14(10):e70353. doi: 10.1002/ece3.70353 (PMC11445282; doi:10.1002/ece3.70353)
Supplement: Supplementary file 1 — TABLE S1. The basic plastomes information of 12 Tamarix samples. [file ECE3-14-e70353-s001.docx]

| **Sample** | **Genbank No.** | **species** | **Collect number** | **Collector** | **Position** | **Date** | **CDS** | **rRNA** | **tRNA** | **Gene number** | **CPG length** | **LSC** | **SSC** | **IR** |
| --- | --- | --- | --- | --- | --- | --- | --- | --- | --- | --- | --- | --- | --- | --- |
| ENC850343 | OR619632 | *Tamarix androssowii* | PE 01177239 | Xinjiang Comprehensive Survey Team | Xingjiang, China | 1959.05.13 | 61 | 2 | 25 | 88 | 128528 | 64583 | 16021 | 23963 |
| ENC850344 | OR619629 | *Tamarix aphylla* | PE 01177243 | C.M.Wang | Taiwan, China | 1998.09.03 | 76 | 8 | 36 | 120 | 156079 | 85060 | 17871 | 26575 |
| MN726883 | MN726883 | *Tamarix ramosissima* | - | - | - | - | 79 | 8 | 36 | 123 | 156138 | 84783 | 18249 | 26554 |
| ON620260 | ON620260 | *Tamarix ramosissima* | - | - | - | - | 79 | 8 | 36 | 123 | 155977 | 84790 | 18249 | 26470 |
| ON920701 | ON920701 | *Tamarix karelinii* | - | - | - | - | 79 | 8 | 36 | 123 | 156170 | 84793 | 18249 | 26565 |
| ON620259 | ON620259 | *Tamarix arceuthoides* | - | - | - | - | 79 | 8 | 36 | 123 | 156180 | 84803 | 18249 | 26565 |
| ENC850351 | OR619631 | *Tamarix jiniperina* | PE 01983734 | S.Y. Lü | Taiwan, China | 1985.06.15 | 79 | 8 | 36 | 123 | 156168 | 84790 | 18250 | 26565 |
| ENC850348 | OR619630 | *Tamarix gracilis* | PE 02068788 | I.I.Rusanovich | Russia | 1983.05.01 | 79 | 8 | 36 | 123 | 156144 | 84745 | 18257 | 26572 |
| MW125612 | MW125612 | *Tamarix taklamakanensis* | - | - | - | - | 79 | 8 | 36 | 123 | 156177 | 84778 | 18259 | 26571 |
| MN229512 | MN229512 | *Tamarix chinensis* | - | - | - | - | 77 | 8 | 36 | 121 | 156190 | 84790 | 18259 | 26571 |
| MK397902 | MK397902 | *Tamarix chinensis* | - | - | - | - | 85 | 8 | 37 | 121 | 156170 | 84767 | 18250 | 26578 |
| ON920700 | ON920700 | *Tamarix laxa* | - | - | - | - | 79 | 8 | 36 | 123 | 156159 | 84777 | 18262 | 26561 |
